# Editorial: Advances in Youth Bullying Research

**DOI:** 10.3389/fpsyg.2022.860887

**Published:** 2022-06-07

**Authors:** H. Colleen Sinclair, Khirsten J. Wilson, Megan Stubbs-Richardson

**Affiliations:** ^1^Department of Psychology, Social Science Research Center, Mississippi State University, Starkville, MS, United States; ^2^Social Science Research Center, Mississippi State University, Starkville, MS, United States

**Keywords:** bullying, adolescence, prevention, cross-cultural, child psychology, aggression

Bullying amongst youth is a worldwide concern. Globally, as many as 246 million children reported experiencing bullying and school violence annually [United Nations Educational Scientific Cultural Organization (UNESCO), 2019]. In the UNESCO report, 32% of children reported bullying victimization with the most common type being psychological or verbal aggression. This special issue highlights the prevalence as well as some of the predictors and buffers of types of bullying occurring among youth from a variety of countries. Specifically, this issue can speak to bullying concerns in Peru, China, Chile, Portugal, Spain, Poland, Russia, Mexico, and the United States.

## Variation in Youth Bullying Statistics

Rates vary across samples in the present special issue. For example, contrasting current bullying statistics in the U.S. where bullying and other forms of victimization appear to be on the decline (Musu-Gillette et al., 2018), researchers have found that the bullying prevalence in Peru has increased, as has the social, emotional, and behavioral impacts of victimization (Arhuis-Inca et al.). Also, in contrast to North American samples, where ~20% of students report bullying victimization, lower rates—16%—were reported in a Russian sample of 6,249 students (Avanesian et al.). These contributions alone demonstrate the importance of examining cultural differences in bullying.

## Consequences of Youth Bullying Behavior

All authors recognized the significant harms of bullying. Research by Peng et al. examined these potentially devastating consequences. In their study of 4,241 7th to 12th grade students in China, they examined the relationship between bullying and self-harm. Their results indicated that different forms of bullying (physical, relational, verbal, and cyber) are associated with different harmful behaviors (self-harm, suicide attempts, and suicidal ideation). Most forms of bullying—except verbal—posed a significant risk for suicide attempts (Peng et al.). This is particularly troubling as the World Health Organization reports suicide as the fourth leading cause of death in 15–19-year-olds worldwide (World Health Organization, 2021), and rates appear to be rising (Zohuri and Zadek, 2020).

## Individual Differences Associated With Bullying Perpetration

Understanding the individual difference variables that affect the experience of bullying and responses to bullying can help guide the implementation of more effective intervention strategies. Zhang et al. conducted a study of 1,631 middle and high school students, analyzing individual differences (i.e., Big Five personality, loneliness, and self-concept) and their influence on bullying behaviors via self-report measures. The links between personality variables and bullying behavior was mediated by loneliness, thus indicating the importance of addressing relational variables as a route for intervention.

Indeed, relationship variables were identified as important in other studies featured in this issue. For instance, Vagos and Carvalhais surveyed 375 youth between 15 and 19 years old to examine the relationship between their attachment quality with parents/peers and their likelihood of engagement in aggressive vs. prosocial behaviors. They found that peer attachment had an indirect effect on prosocial behaviors and quality maternal relationships indirectly resulted in a decrease in overt aggression and delinquency.

Stubbs-Richardson et al. tested the Multimotive Model (MMM), which measures prosocial, asocial, antisocial responses to bullying victimization in a sample of 605 American high school students. Relational variables were key to predicting whether victimized youth would choose a prosocial path over an antisocial response. Students who perceived having fewer supportive relationships were least likely to choose prosocial responses. Relatedly, those seeking help when bullied were more likely to report strong peer connections and family communication (Sitnik-Warchulska et al.).

Further research in Silesia is consistent with this relational narrative. Children engaging in bullying perpetration often reported low quality parental relationships (Sitnik-Warchulska et al.). Low quality family relationships were also linked to bullying victimization as revealed by a study of 2,415 Mexican youth (9–15 years old), where familial child abuse (i.e., emotional, physical, and sexual) was strongly linked to peer victimization (i.e., direct, indirect, and cyberbullying; Martin-Babarro et al.).

## Impact of School Climate on Youth Bullying

Youth bullying is also embedded in school culture. Researchers examining the validity of the Dual School Climate and School Identification Measure—Student (SCASIM-St15) in 2,044 Chilean school-aged children found that negative school climate is associated with tolerance of antisocial behavior (Gálvez-Nieto et al.). Students who hold a more positive perception of their school climate were less likely to break the rules; those who positively identify with their school may view authority figures more positively and thus be more willing to seek help.

Other aspects of school climate included examined how much students perceived having help and how well-equipped they were to deal with bullying. A study consisting of 75 Silesian students analyzed the relationship between the probability of help-seeking behaviors and bullying risk factors (Sitnik-Warchulska et al.). They found that the majority of participants exhibited help-seeking behaviors, most of which was directed toward family followed by peers (Sitnik-Warchulska et al.). This perceived presence of support proved to be vital in reducing bullying prevalence in schools.

Researchers in Russia, consistent with past work on school climate variables, noted that “Bullying…tends to develop more frequently in a competitive environment” (Avanesian et al., p. 1). They encourage schools to foster a less competitive context to decrease bullying.

In another study by Montero-Carretero et al. of 629 Spanish students between the ages of 12 and 14 years old examined the relationship between school climate and bullying behaviors. Results indicated that when students perceived greater teacher support and rule clarity, they experienced more positive perceptions of school climate and lower levels of victimization (Montero-Carretero et al.). Thus, across cultures, various improvements in school climate appear promising for reducing the harm of bullying, if not reducing the bullying behavior itself.

## Additional Considerations for Interventions

Some of the papers featured herein tested specific interventions whereas others identified additional routes for intervention beyond what was discussed above. For example, researchers examining the effects of the “Zero Violence Brave Club” as a prevention effort among young children found that, after its implementation, participants became more aware of the problem, were less favorable toward aggression, valued kindness, and increased bystander peer intervention (Roca-Campos et al.). Compellingly, this research involved a diversity of school environments and showed effects across contexts.

Outside of the school walls, Stives et al. point to the importance of interventions involving more than just the children. They examined parental perspectives of bullying. Results showed that most parents find bullying to be problematic but feel that their children under-reported to them about instances of bullying. The researchers recommend the greater inclusion of parents in anti-bullying efforts as there was a strong interest among parents interviewed in addressing the problem.

Beliefs about bullying are also influenced by societal values. When examining the relationship between Belief in a Just World (BJW) Hypothesis and responses to bullying, researchers found that higher global BJW, instead of personal BJW, was correlated with minimization of perpetrator actions (Voss and Newman). Therefore, belief in a just world may constitute victim blaming which is counterproductive to bullying prevention efforts. Thus, countering these attitudes could be means to improve assistance afforded to victims.

## Conclusion

What is consistently shown, no matter the cultural context, is that bullying hurts, carrying significant negative outcomes for bullies, victims, and bully-victims. Research evidence collected here also revealed factors that may be helpful for intervention purposes. Namely, the research shows how changes to school climate—such as reducing competition—and the involvement of the community—such as parents and peers—can reduce the impact of bullying and bullying prevalence, as well as enhance prosocial behavior. In particular, the importance of social connection for curbing antisocial behavior was a consistent theme cross-culturally. As bullying is a worldwide problem it requires cross-cultural research to address the associated problems and outcomes. The present special issue addresses this need.

## Author Contributions

KW focused on article summaries. MS-R on the introduction, fact-checking, and references. HS on conclusion, editing of the entirety, checking against results, cutting to meet word limits, refinement, and flow. All author contributed to the writing, editing, summaries, and fact-checking of the material within this manuscript.

## Conflict of Interest

The authors declare that the research was conducted in the absence of any commercial or financial relationships that could be construed as a potential conflict of interest.

## Publisher's Note

All claims expressed in this article are solely those of the authors and do not necessarily represent those of their affiliated organizations, or those of the publisher, the editors and the reviewers. Any product that may be evaluated in this article, or claim that may be made by its manufacturer, is not guaranteed or endorsed by the publisher.
